# Metadynamic Recrystallization in the Isothermal Double Compression of CP800 Steel

**DOI:** 10.3390/ma18071549

**Published:** 2025-03-29

**Authors:** Xiaoyu Yang, Zhenli Mi, Wangzhong Mu

**Affiliations:** 1 Institute of Engineering Technology, University of Science and Technology Beijing, Beijing 100083, China; b20190509@xs.ustb.edu.cn; 2 Engineering Materials, Department of Engineering Science and Mathematics, Luleå University of Technology, 97187 Luleå, Sweden

**Keywords:** metadynamic recrystallization, softening behavior, CP steel, isothermal double compression

## Abstract

The global drive toward decarbonization has spurred industry interest in the compact steel production (CSP) process for manufacturing automotive steel sheets. Understanding the hot deformation behavior, particularly the metadynamic softening mechanism occurring between passes, is essential for evaluating process feasibility under CSP process. This study investigates the metadynamic softening behavior of CP800 steel intended for CSP applications, utilizing isothermal double compression tests performed at the deformation temperatures of 1173, 1273, and 1373 K, strain rates of 0.1,1, and 5.0 s^−1^, and the interpass times of 1, 10, and 20 s. The softening behavior was assessed through the deformation flow stress–strain curves under varying conditions, and a kinetic equation of metadynamic recrystallization was proposed and validated against experimental data. Additionally, the effect of initial austenite grain sizes of 42 μm and 92 μm on metadynamic recrystallization were analyzed. Results indicate that the final rolling pass temperature should exceed 1173 K to prevent mixed grain structures. Although grain refinement induced by the metadynamic recrystallization in CP800 steel was found to be independent of initial grain size, the final grain size itself remained sensitive to the initial grain dimensions. Adopting lower holding temperature and shorter holding durations prior to rolling is advisable for energy-efficient CSP, provided compositional homogeneity and suitable deformation temperatures are maintained. These insights contribute valuable guidance for optimization CSP process in the production of CP800 steel.

## 1. Introduction

Given that steel manufacturing contributes approximately 30% of global industrial CO_2_ emissions [1], replacing conventional steel production technology with energy-efficient processes is crucial for achieving sustainable decarbonization targets. Compact strip production (CSP) technology, a thin slab, near-net-shape forming technology, integrates continuous thin-slab casting with direct strip hot rolling [2], significantly reducing energy consumption and associated CO_2_ emissions due to the elimination of the reheating process prior to hot rolling [3]. Hot-rolled low-carbon steels and high-strength low-alloy (HSLA) steels have already dominated CSP market supplication for decades [4]. In recent years, there has been increasing interest in producing steels grades with higher alloy compositions and complex phases via the CSP process, owing to the technology’s notable chemical and microstructural homogeneity in the CSP slab [5].

Advanced High-Strength Steels (AHSSs), particularly complex phase (CP) steels, have gained prominence in automotive applications [6], demonstrating significant potential for CSP technology. Given CSP’s distinctive processing features, extensive research efforts have been directed toward metallurgical design, microstructure evolution, and property consistency optimization of AHSS products [7,8,9,10,11,12]. Nevertheless, grain size heterogeneity remains a critical issue within CSP-produced steel. Zhu et al. [13] attributed this grain size variability to insufficient homogenization, resulting from the omission of reheating processes. Similarly, Dong et al. [14] indicated that inappropriate temperature control and deformation scheduling in Nb micro-alloyed CSP strips exacerbate grain structure heterogeneity. Consistent with these perspectives, DeArdo et al. [15] and Romano-Acosta et al. [16] suggested that elevating deformation temperatures to recrystallization-limit temperature (T95) effectively reduces mixed grain structures. Although these studies have highlighted the underlying issues and proposed empirical solutions, a universally applicable and precise guideline for optimizing CSP processing conditions has yet to be established.

The establishment of the recrystallization kinetic equation is a widely accepted mathematical approach to modeling the austenite evolution during hot deformation, thereby addressing related process requirements. Compared to the conventional rolling process, CSP technology features fewer rolling passes, resulting in higher reductions per pass with generally lower strain rates [17]. In addition, due to the compact arrangement of CSP rolling stands, the interpass times are shorter, insufficient for nucleation required by static recrystallization [18]. Consequently, metadynamic recrystallization, rather than static recrystallization, is more likely to take place in the subsequent interpass periods following dynamic recrystallization in the CSP process [19]. However, little information is available regarding metadynamic recrystallization, either in behavior or recommended applications. Some works only focused on the effect of hot deformation parameters on dynamic recrystallization, neglecting the tint of the important meaning of metadynamic softening between passes [20,21]. Liu et al. [22] proposed a well-fitting kinetics equation describing the metadynamic recrystallization behavior in 300M steel, but did not thoroughly explore its implications for processing optimization. Tang et al. [23] investigated the recrystallization behavior during the CSP process in 510L steel, although the deformation temperatures were lower than those typically used for AHSS production. Other works have combined laboratory experiments and trial production in high-carbon bainitic steel [24] and Fe-Mn-Al-C steel [25], providing valuable insight into the semi-industrial mathematical predicted models. Hence, a foundation of metadynamic recrystallization behavior specific to CSP process is necessary to fill existing research gaps and facilitate the application of CSP technology in AHSSs.

In this study, the metadynamic softening behavior of a CP800 steel designed for the CSP applications was investigated through isothermal double compression. The softening behaviors were discussed through the flow stress–strain curves under varying deformation conditions. The kinetic model describing the metadynamic recrystallization was proposed and validated experimentally. Furthermore, a comparative analysis involving initial austenite grain size was performed to elucidate their effects on grain refinement. The findings provide deeper insights into the metadynamic softening behavior of CP800 steel, offering practical recommendations for optimizing CSP processes.

## 2. Materials and Methods

The experimental material used in this study was CP800 steel manufactured by continuous casting with the composition (wt.%) of 0.05C-0.25Si-1.5Mn-0.12Ti-0.4Cr-Fe. The Ac_3_ temperature of CP800 steel is 1189 K, according to the rate of 10 K/s heating condition dilatometric measurements with a diameter of 4 mm × 10 mm length cylinder.

**Figure 1:**
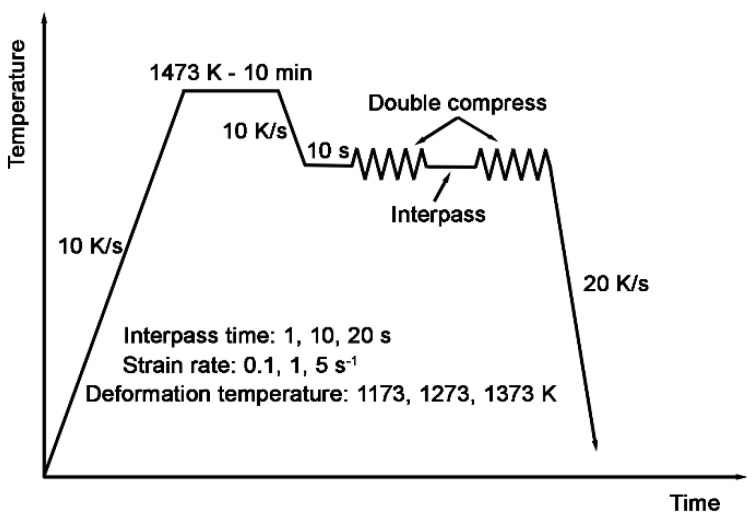
Schematic illustration of the double isothermal compression process.

Compression experiments were carried out on the Gleeble 3500 thermal simulation system using the cylindrical samples (φ10 mm × 15 mm), with graphite lubricant applied at both ends of the cylinder to reduce the effect of friction during compression. Samples were heated at 10 K/s from room temperature to 1473 K and held for 10 min to homogenize the chemical composition and uniform the austenite grain size. Subsequently, samples were cooled at 10 K/s to the compress deformation temperatures and hold at a isothermal condition for 10 s for the stabilization of the deformation condition. Next, the compression was conducted at deformation temperatures of 1173 K, 1273 K, and 1373 K, with strain rates of 0.1 s^−1^, 1 s^−1^, and 5 s^−1^, and a strain of 0.5, exceeding the value of critical strain of dynamic recrystallization and ensuring the metadynamic recrystallization occurs between passes. The specimens were held for interpass times of 1 s, 10 s, and 20 s before the isothermal second compression. After double compression, the specimens were rapidly cooled at rates of around 20 K/s to preserve the austenite grain size through blow air in the chamber. The process is detailed illustrated in Figure 1. More than 3 parallel samples were tested in each deformation condition to ensure the reliability of the data.

Metallographic samples for grain size statistically were cut from the middle of deformed specimens. Samples were ground under 400, 800, 1000, 1200, 1500, and 2000 grit SiC sandpaper, then mechanically polished using diamond polishing paste with a granularity of 1.0 μm, and finally etched in 4% Nital solution (4% nitric acid, 96% ethanol). The final microstructures were captured by a laser confocal microscope (OLS4100, Optical Imaging Systems, Beijing, China), and average grain size was quantified by Image J (1.54g with Java 1.8.0) software according to the line intercept methods [26]. More than 5 regions per sample were analyzed to ensure the measurement accuracy.

## 3. Results and Discussion

### 3.1. Flow Stress–Strain Behavior in CP800 Steel

**Figure 2:**
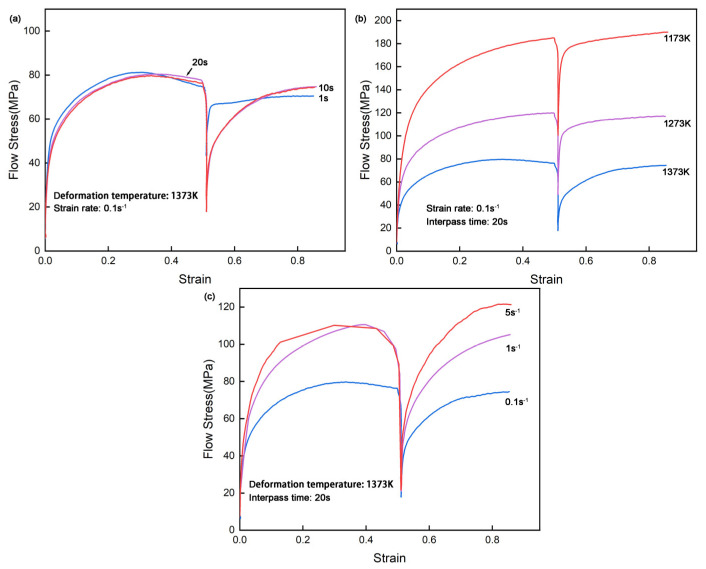
Flow stress–strain curves in the double isothermal compression of CP800 steel at different (**a**) interpass times, (**b**) deformation temperatures, and (**c**) strain rates.

Figure 2 presents the flow stress–strain curves of CP800 steel obtained from double isothermal compression under varying interpass times, deformation temperatures, and strain rates. The yield flow stress in the first compression of each curve is consistently lower than that in the second compression. It is suggested that since a higher density of dislocations is introduced during the first compression, a higher stress is required in the second compression to overcome the resistance of movable dislocations [27,28].

It can be clearly seen in Figure 2a that the second yield stress decreases, and the softening effect becomes more pronounced as the interpass time extends from 1 s to 10 s. The interpass time of 1 s is insufficient for complete recrystallization of the dislocations and grains inside the material. In addition, the compression curves of 10 s and 20 s interpass times overlap, which means a consistent and fully metadynamic softening occurs between the interpass. In other words, the metadynamic recrystallization should finish within 10 s under compress conditions of 1373 K and a 0.1 s^−1^ strain rate. This also implies that any incremental grain growth occurring during this additional 10 s has a negligible impact on subsequent deformation behavior. It is also known from Figure 2a that the peak stress during the second compress is slightly lower than that in the first pass, which can be attributed to the dynamic recrystallization in the first pass judging from the profile of the curve. The nucleation associated with dynamic recrystallization in the first compression effectively reduces the dislocation density, resulting in the observed reduction in peak stress of the second pass [22,29].

Figure 2b demonstrates that the second flow stress is slightly lower than that of the first pass, with this decreasing trend becoming more pronounced as deformation temperature increases from 1173 K to 1373 K. This phenomenon can be attributed to the progressively softening effect of metadynamic recrystallization and the increased softening rate of the material [30,31]. Figure 2c reveals that CP800 is prone to go through a highly metadynamic recrystallization under a 5 s^−1^ strain rate than under 0.1 s^−1^, according to the second peak stress. When grains are nearly fully recrystallized between passes, smaller grain size and reduced dislocation density lead to increased deformation resistance, resulting in elevated second peak stress. It is reasonable to say that the softening driving force is positively correlated with increased strain rates, governed primarily by the dislocation behavior. Moreover, the flow stress declines with rising deformation temperatures shown in Figure 2b and decreasing strain rate in Figure 2c, reflecting the material’s inherent properties, including work hardening behavior in different temperatures or strain rates.

### 3.2. Process Effect on the Metadynamic Softening of CP800 Steel

The quantification of the metadynamic softening behavior between passes enhances the understanding of the high-temperature compression characteristics of CP800 steel. The experimental-based calculation can be adopted by the 0.2% offset-stress method [32,33]. The metadynamic softening fraction (F_s_) is defined as follows:

(1) $$F_{s}=\frac{\sigma_{m}-\sigma_{2}}{\sigma_{m}-\sigma_{1}}$$

where σ_m_ is the flow stress at the end of the first deformation, and σ_1_ and σ_2_ are the yield stresses determined at an offset strain of 0.2% for the first and second compression, respectively. In addition, the effect of adiabatic heating is neglected in this work due to measurement limitations and relatively low strain rate sensitivity of steel grades investigated.

**Figure 3:**
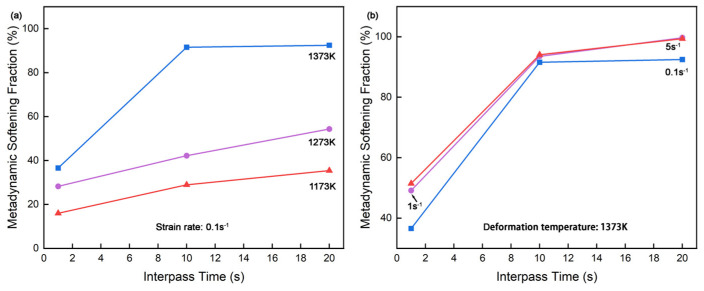
Effect of (**a**) the deformation temperature and (**b**) strain rate on the metadynamic softening of CP800 steel.

Figure 3a shows the metadynamic softening fraction of CP800 steel at the strain rate of 0.1 s^−1^ varied with the deformation temperatures and interpass time. The fraction of metadynamic softening gradually increased from 16%, 29%, and 35% followed by the increasing interpass time on 1173 K. The similar upward trending is observed in 1273 K (28%, 42%, and 54%) and 1373 K (37%, 91%, and 92%) curves, with nearly complete softening achieved at 1373 K at 10 s and 20 s interpass time, consistence with the analysis on Figure 2a. It seems the fraction of metadynamic softening clearly depends on interpass time, yet only around 35% at a deformation temperature of 1173 K and an interpass time of 20 s. The climbing trend goes gently along with the interpass time increase in the 1173 K and 1273 K curves, which is reasonable considering that the full metadynamic softening cannot occur at these deformation conditions. This strongly supports the notion that metadynamic recrystallization is a thermally activated process, as increased temperature facilitates atomic vibrations and reduces the atomic-binding forces, enhancing dislocation mobility and recrystallization kinetics [34]. Additionally, the increased softening fraction with higher deformation temperature at fixed interpass time confirms the significant influence of temperature on metadynamic softening rate.

The relationship between strain rate and the metadynamic softening fraction at 1373 K is displayed in Figure 3b across different interpass times. More grains are going through metadynamic softening at higher strain rates of 1 s^−1^ and 5 s^−1^ rather than 0.1 s^−1^. Although the soften fraction at a strain rate of 1 s^−1^ averages about 2% less than 5 s^−1^ at interpass time 1 s, both conditions reach nearly identical values at the interpass time of 10 s of 94% and 20 s of 99%. CP800 could fully recrystallize during passes under this deformation condition. In contrast, at the strain rate of 0.1 s^−1^, the metadynamic softening fraction could only reach about 92%, signifying the difficulty of the metadynamic recrystallization in a lower strain rate such as 0.1 s^−1^ even with prolonged interpass time, not to mention under lower deformation temperatures. Higher strain rates inherently introduce dislocations in steels [35,36] which could be proven by the elevated stress in the first compression of the 5 s^−1^ stress–strain curve shown in Figure 2c. The resulting high density of dislocations provides sufficient energy, enhancing the driving force for metadynamic recrystallization by facilitating the competition between metadynamic softening and work hardening.

### 3.3. Kinetic Study of the Metadynamic Recrystallization of CP800 Steel

The recrystallization is usually assumed to start when the softening fraction F_s_ = 0.2 [37], and the fraction of metadynamic recrystallization (X_m_) is determined as follows:

(2) $$X_{m}=\frac{F_{s}-0.2}{1-0.2}=\frac{F_{s}-0.2}{0.8}$$

The classic Avrami equation [38,39,40] is employed to analyze the kinetic behavior of metadynamic recrystallization in this study. The equation is defined as follows:

(3) $$X_{m}=1-\exp\left[-0.693{\left(\frac{t}{t_{0.5}}\right)}^{n}\right]$$

where t is the interpass time (s), t_0.5_ is the time (s) for 50% completion of metadynamic recrystallization, and n is the constant parameter dependent on the material. The above formula shows that the metadynamic recrystallization kinetics are predominantly influenced by t_0.5_ and n, which is evident when expressed in logarithmic form as shown in Equation (3):

(4) $$\ln\left[\ln\left(\frac{1}{1-X_{m}}\right)\right]=\ln0.693+n\ln t-n\ln t_{0.5}$$

**Figure 4:**
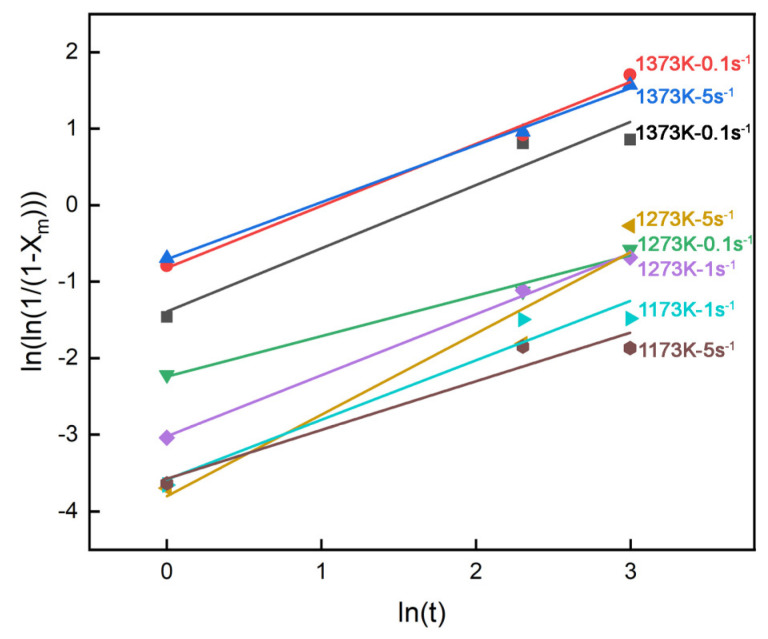
Relationship between ln[ln(1/(1 − X_m_))] and lnt in different temperatures and strain rates.

The value of n was obtained through linear fitting by plotting ln[ln(1/(1 − X_m_))] versus lnt, as illustrated by Figure 4, and n is calculated as an averaged value of 0.77. The value of t_0.5_ was determined from the intercept obtained by linear regression analysis according to Figure 4.

Another decisive parameter $t_{0.5}$ could be expressed as follows:

(5) $$t_{0.5}=A{\dot{\varepsilon }}^{r}\exp\left(\frac{Q}{\mathrm{RT}}\right)$$

where $\dot{\varepsilon }$ is the strain rate, Q is the activation energy (J/mol) for metadynamic recrystallization, R is the mole gas constant which is equal to 8.314 J/K·mol, T is the deformation temperatures (K) when recrystallization occurs between passes, and A and r are the material dependent constants. The values of A and r could also be obtained by fitting followed by Equation (5) after applying the natural logarithm:

(6) $$\ln t_{0.5}=\ln A+r\ln\dot{\epsilon }+\frac{Q}{\mathrm{RT}}$$

**Figure 5:**
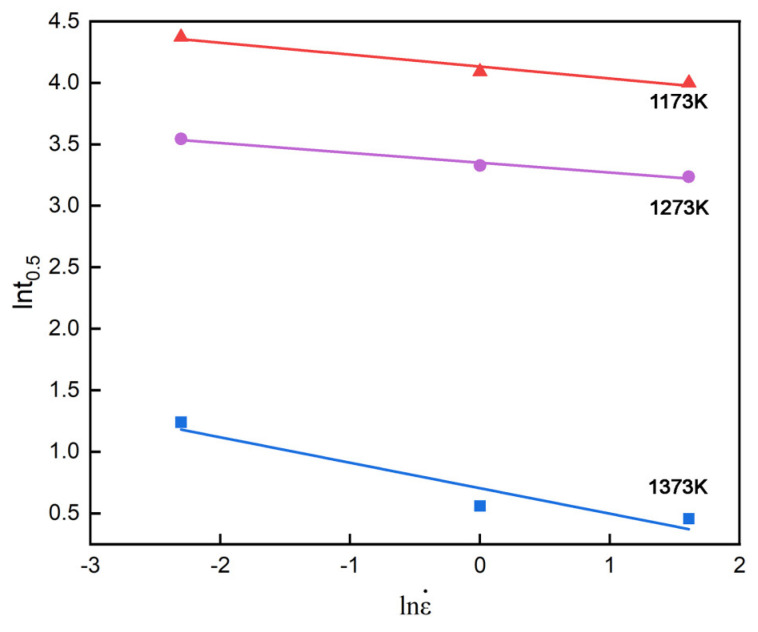
Relationship between and $\ln\dot{\varepsilon }$ and $\ln t_{0.5}$ in different temperatures.

Figure 5 displays the relationship between ln$\dot{\epsilon }$ and lnt_0.5_ along with the corresponding linear fit, which can identify the value of r equal to the slope as −0.13 on average.

**Figure 6:**
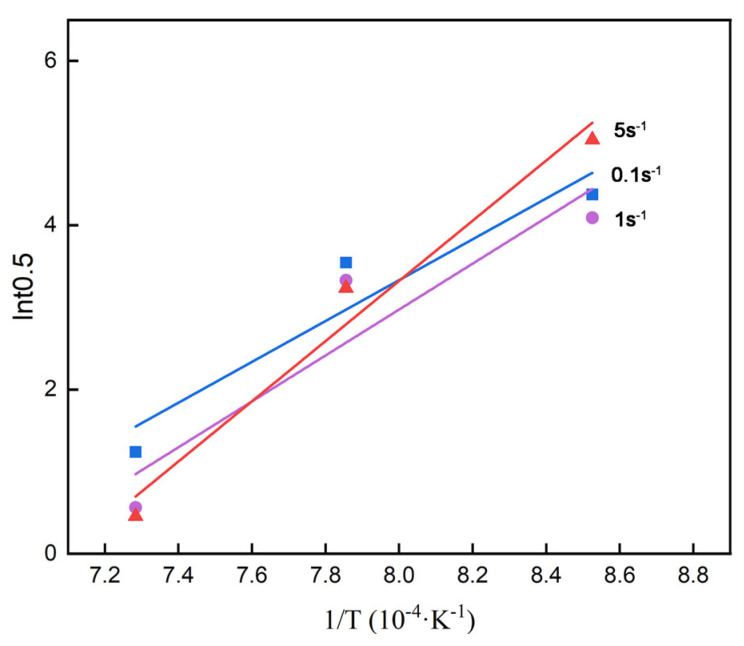
Relationship between and 1/T and $\ln t_{0.5}$ in different temperatures.

The value of Q was addressed as 247,930 J/mol through the fitting curves of 1/T and lnt_0.5_ shown in Figure 6. As we can see, the value of Q is slightly higher than that in low-carbon steels reported previously [22,23]. Potential mathematical errors due to averaging during linear fitting should be acknowledged. In addition, the precipitation effects and solute drag effects from alloying elements such as Mn, Ti, and Cr in CP800 steel likely affect the recrystallization kinetics, explaining the similarity in Q value to other alloying steel [41,42]. Finally, the constant A can be achieved as 1.08 × 10^−9^ based on the above calculation.

The kinetic behavior of the metadynamic recrystallization of CP800 steel described by Equations (3) and (5) is presented as follows:

(7) $$X_{m}=1-\exp\left[-0.693{\left(\frac{t}{t_{0.5}}\right)}^{0.77}\right]$$

(8) $$t_{0.5}=1.08\times 10^{-9}{\dot{\varepsilon }}^{-0.13}\exp\left(\frac{247930}{\mathrm{RT}}\right)$$

**Figure 7:**
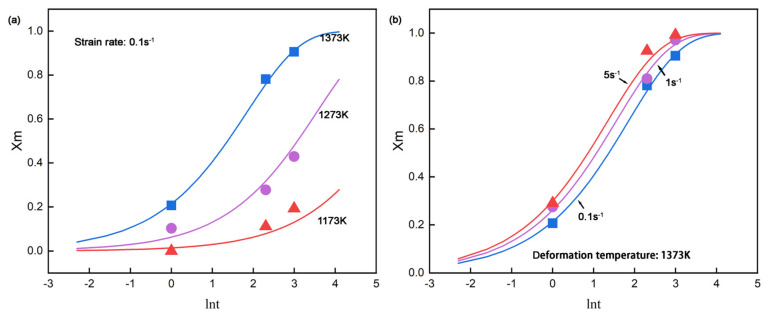
The comparison of the experimental (scatted dots) and the simulation (lines) metadynamic softening fraction at (**a**) different deformation temperatures and (**b**) strain rates.

The comparison of experimental results and calculation fitting according to Equations (7) and (8) is shown in Figure 7. It seems that the results match well with the predicted values, with maximum and average deviations of 6.2% and 2.3% in metadynamic recrystallization fraction. The established kinetic equations for CP800 steel thus provide a reliable basis for qualitative semi-empirical analysis and fundamental process design. From the fitted profiles of X_m_ presented in Figure 7a, complete metadynamic softening is evident for interpass time exceeding 10 s at 1373 K across all strain rates. In contrast, it seems the metadynamic softening was hardly carried out at 1173 K under most conditions even given a longer interpass time, suggesting that finishing rolling at temperatures above 1173 K is advisable to prevent undesirable mixed grain structures. Hence, rolling parameters, including the number of rolling passes, roll spacing, rolling speed, etc., need to be carefully considered to meet the suggestions.

### 3.4. Initial Grain Size Effect on the Metadynamic Softening of CP800 Steel

**Figure 8:**
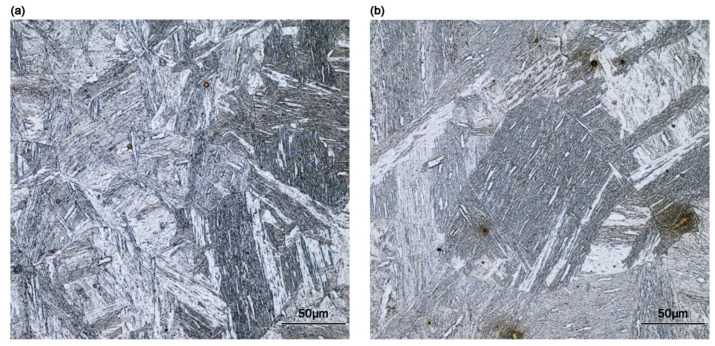
Direct quenching OM microstructures of CP800 steel in (**a**) holding 10 min at 1473 K and (**b**) holding 30 min at 1473 K.

In the compact steel production process, the temperature variations commonly occur between the head and tail of continuous casting billet before rolling, primarily due to shortened or insufficient holding times. This leads to the differences in initial austenite grain size before rolling deformation. Hence, two distinct initial austenite grain sizes were constructed in this study to identify the effect of initial grain size on metadynamic recrystallization: 42 μm and 92 μm initial austenite grains were produced by 10 min and 30 min isothermal holding at 1473 K, respectively, before isothermal double compression. Microstructures after directly quenching from these isothermal holding conditions are shown in Figure 8.

**Figure 9:**
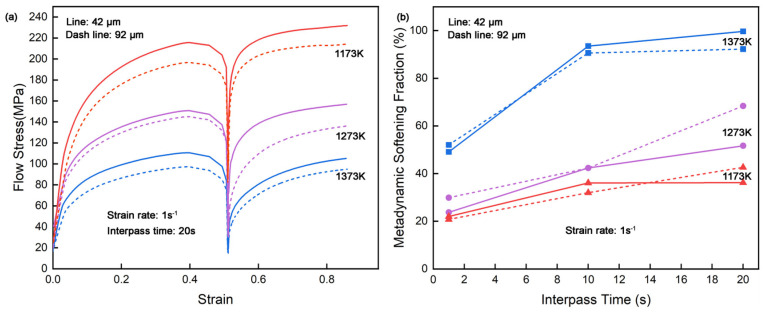
Metadynamic recrystallization behavior of CP800 steel in 42 μm and 92 μm initial austenite grains, (**a**) flow stress–strain curves in the double isothermal compression at different deformation temperatures, and (**b**) effect of the deformation temperature on the metadynamic softening.

The flow stress–strain curves and metadynamic softening fraction at 1 s^−1^ strain rate of different initial grain sizes are presented in Figure 9. From Figure 9a, samples with 92 μm initial austenite grains exhibits lower flow stresses compared to those with the 42 μm samples, reflecting the influence of grain refinement strengthening. The fundamental deformation behavior such as the relationship between deformation temperatures on flow stress, or between interpass time and metadynamic softening fraction, remains consistent regardless of the initial grain size, no matter the deformation conditions. Figure 9b also further illustrates a negligible difference in the metadynamic softening fraction between the two grain sizes, indicating that the metadynamic softening fraction of CP800 steel is relatively insensitive to the initial grain size. This observation aligns with the explanation by Sun et al. [37], who suggested that the extent of metadynamic recrystallization that occurred in the first compression does not significantly vary with grain size, resulting in similar softening behaviors during interpass intervals.

### 3.5. Grain Size After Metadynamic Softening of CP800 Steel

**Figure 10:**
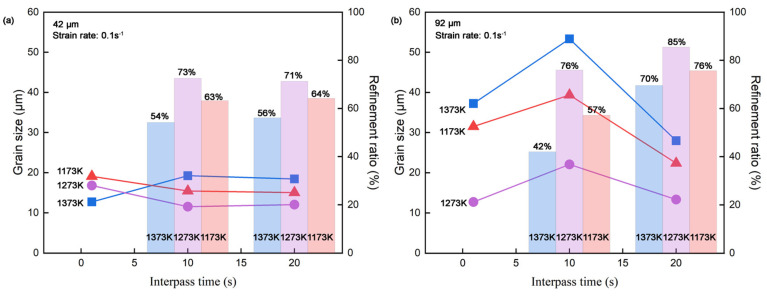
Grain size after metadynamic softening curves and refinement ratio bar of CP800 steel at 0.1 s^−1^ strain rate in 1, 10, and 20 s interpass times of (**a**) 42 μm and (**b**) 92 μm initial austenite grains.

**Figure 11:**
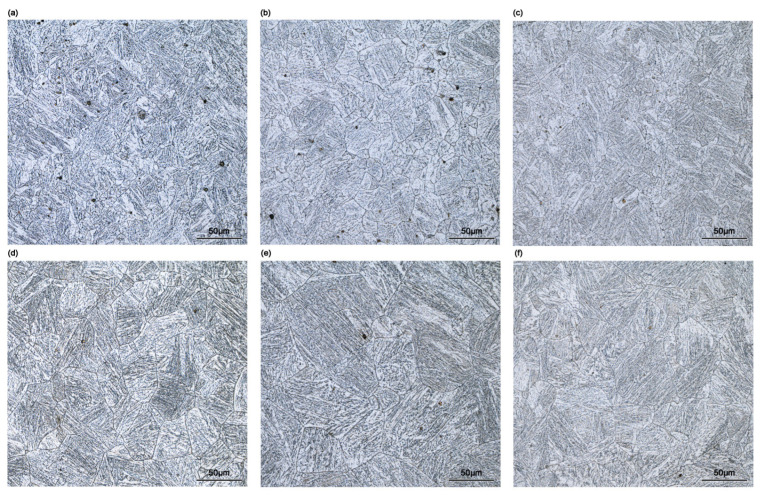
OM microstructures of CP800 steel in (**a**–**c**) 42 μm and (**d**–**f**) 92 μm initial austenite grains in 1473 K, 0.1 s^−1^, and interpass times of (**a**,**d**) 1 s; (**b**,**e**) 10 s; and (**c**,**f**) 20 s.

Grain size after 0.1 s^−1^ strain rate deformation for samples with initial austenite grain sizes of 42 μm and 92 μm are performed in Figure 10. OM microstructures obtained under the deformation condition of 1373 K and 0.1 s^−1^ for both initial grains are shown in Figure 11. As we can see from Figure 10a, the 1173 K line shows a typical refining trend due to metadynamic recrystallization, with grain refinement becoming more pronounced as interpass times increases, consistent with observations in Figure 3a and Figure 7a. The trend of the 1273 K line is the same as the 1173 K line in the first part of Figure 10a, while slight grain coursing appears after 20 s softening, likely resulting from grain growth due to prolonged isothermal time. At 1373 K, the initial grain refinement observed at short interpass times (1 s) is more significant compared to lower temperatures, as confirmed by OM microstructures in Figure 11b, where the formation of numerous fine recrystallized grains contributes to the reduction in average grain size value.

This phenomenon was also observed in 92 μm initial austenite grains samples shown in Figure 10b, indicating significant recrystallization within a short interpass time (Figure 11d,e). The refinement ratio (%) was calculated as $100\;\times \;(D\;-\;D_{0})/D_{0}$, where D is the average diameter after compression, and D_0_ is the initial austenite grain size. Since the average grain size measured after 1 s interpass exhibited significant heterogeneity, only refinement ratios for interpass times of 10 s and 20 s are shown in Figure 10. The samples with 42 μm initial austenite grains reached stable refinement after 10 s interpass time, showing little change at 20 s. In contrast, the 92 μm initial austenite grains samples continued refining up until 20 s (Figure 11f), consistently showing higher refinement ratios than 42 μm initial austenite grains samples in all conditions (Figure 10). It should be noted that in the experimental group with a 20 s interpass time, manual size counting might overestimate the grain refinement of metadynamic recrystallization, due to the inclusion of newly nucleated grains from potential static recrystallized. However, the results of softening tendency remain valid. The most efficient deformation temperature is 1273 K at the strain rate of 0.1 s^−1^ in both initial austenite grain samples, providing guidance for rolling temperatures optimization during production. Regarding final recrystallization grain size, the 42 μm initial austenite grains samples present finer microstructures (Figure 11c,f). Hence, maintaining slabs at higher temperatures or for longer time to achieve bigger initial grain size before rolling is unnecessary, provided chemical composition homogeneity is ensured. This strategy supports energy efficiency and reduces processing time. Although the manufacturing involves greater complexity than the laboratory condition, the insights derived from this study draws an optimization script and is highly trustworthy for the procedure recommendation.

## 4. Conclusions

In this study, the metadynamic recrystallization behavior of CP800 steel was investigated through the isothermal double compression at deformation temperatures of 1173, 1273, and 1373 K, and at strain rates 0.1, 1, and 5 s^−1^, under different interpass times of 1, 10, and 20 s. The effect of initial grain size in 42 μm and 92 μm on metadynamic recrystallization was also discussed. The conclusions are in the following:

1. The metadynamic recrystallization fraction increases with rising deformation temperature, strain rate, and interpass time. At 1373 K deformation, the metadynamic softening occurs sufficiently at 10 s interpass time under all experimental strain rates. It seems the metadynamic softening was hardly carried out at 1173 K under the studied conditions.
2. The fraction of the metadynamic recrystallization of CP800 steel can be accurately represented by the kinetic equation $X_{m}=1\;-\exp\left[-0.693{\left(t/t_{0.5}\right)}^{0.77}\right]$, and the time (s) for 50% metadynamic recrystallization predicted as $t_{0.5}=1.08\times 10^{-9}{\dot{\varepsilon }}^{-0.13}\exp\left(247930/\mathrm{RT}\right)$. The fitting is in good agreement with the experimental results.
3. Under the conditions studied, the initial grain size has minimal influence on the metadynamic softening fraction, although it significantly affects the final recrystallized grain size. The 92 μm initial austenite grains samples have higher refinement efficiency; however, the 42 μm initial austenite grains samples present a smaller size on the final recrystallization grain size. The most efficient deformation temperature is 1273 K among these experimental conditions.
4. Based on the above discussion, it is recommended that the final pass of rolling should be conducted at temperatures above 1173 K to avoid undesirable mixed grains. Additionally, a lower holding temperature and shorter holding time before rolling are advised for compact process and energy saving under the premise of chemical composition homogeneity and final deformation temperature.

## Data Availability

Due to privacy restrictions, we are currently unable to make the data publicly accessible. However, we would like to emphasize that these data can be made available upon reasonable request by contacting the corresponding author, provided that such requests comply with privacy-related guidelines.
